# Supplementary material for: Evaluation of the International Society for Cutaneous Lymphoma Algorithm for the Diagnosis of Early Mycosis Fungoides
Source: Cells. 2021 Oct 15;10(10):2758. doi: 10.3390/cells10102758 (PMC8534563; doi:10.3390/cells10102758)
Supplement: Supplementary file 1 [file cells-10-02758-s001.zip › cells-1372707-supplementary.pdf]

Supplementary Table S1. Clinicopathologic features and ISCL scores of cases included in this study

Case No.	Sex	Age (yrs)	CD2 (%)	CD3 (%)	CD5 (%)	CD7 (%)	Epidermal discordance	TCR-γ	TCR-δ	TCR-β	Total score	Clinical score	Histo-pathologic score	Immuno-pathologic score	Molecular/ biologic score	Clinical diagnosis after ancillary studies
1	Female	46	95	95	95	20	None	WT	WT	WT	1	0	1	0	0	Annular erythema
2	Female	68	80	90	80	50	None	WT	WT	WT	1	0	1	0	0	Drug eruption
3	Male	49	95	95	95	20	None	WT	WT	WT	1	0	1	0	0	Parapsoriasis
4	Male	54	95	95	95	40	None	WT	WT	WT	1	0	1	0	0	Chronic eczema
5	Female	60	100	90	100	60	None	WT	WT	WT	1	0	1	0	0	Pseudolymphoma
6	Male	26	95	95	95	20	None	WT	WT	WT	1	0	1	0	0	Mycosis fungoides
7	Male	60	95	95	95	25	None	WT	WT	WT	1	1	0	0	0	Morphea
8	Female	41	95	95	95	25	None	WT	WT	WT	1	1	0	0	0	Mycosis fungoides
9	Female	77	95	95	95	35	None	WT	WT	WT	2	1	1	0	0	Parapsoriasis
10	Male	69	95	95	95	85	None	WT	WT	WT	2	1	1	0	0	Psoriasiform dermatitis
11	Female	20	95	95	95	30	None	WT	WT	WT	2	0	2	0	0	Mycosis fungoides
12	Female	58	95	95	95	95	None	WT	WT	WT	2	0	2	0	0	Parapsoriasis
13	Female	39	80	80	80	30	None	WT	WT	WT	2	2	0	0	0	Parapsoriasis
14	Male	21	90	90	90	50	None	WT	WT	WT	2	2	0	0	0	Lupus panniculitis
15	Male	14	90	95	90	80	None	Mutated	Mutated	WT	3	1	1	0	1	Mycosis fungoides
16	Female	51	80	95	80	60	None	Mutated	Mutated	WT	3	0	2	0	1	Mycosis fungoides
17	Male	66	95	95	95	35	None	WT	WT	WT	3	1	2	0	0	Parapsoriasis
18	Male	60	90	95	95	50	None	WT	WT	WT	3	2	1	0	0	Parapsoriasis
19	Male	57	90	90	90	20	None	WT	WT	WT	3	1	2	0	0	Mycosis fungoides
20	Female	74	95	95	95	40	None	WT	WT	WT	3	2	1	0	0	Mycosis fungoides
21	Female	72	95	95	95	20	None	Mutated	WT	WT	3	2	0	0	1	Parapsoriasis
22	Female	60	95	95	95	55	None	Mutated	Mutated	WT	3	1	1	0	1	Mycosis fungoides
23	Male	33	90	95	90	40	CD7	Mutated	WT	Mutated	4	2	0	1	1	Mycosis fungoides
24	Female	50	90	95	95	3	None	Mutated	WT	Mutated	4	0	2	1	1	Mycosis fungoides
25	Male	27	80	95	90	5	CD7	Mutated	WT	Mutated	4	1	1	1	1	Mycosis fungoides
26	Male	52	90	90	90	30	None	Mutated	WT	WT	4	2	1	0	1	Mycosis fungoides
27	Male	60	80	90	70	5	None	Mutated	WT	WT	4	2	0	1	1	Mycosis fungoides
28	Male	74	95	95	95	35	CD7	Mutated	WT	WT	4	1	1	1	1	Mycosis fungoides
29	Female	60	95	95	95	55	None	Mutated	Mutated	WT	4	2	1	0	1	Mycosis fungoides
30	Female	65	95	95	95	5	CD7	WT	WT	WT	4	1	2	1	0	Mycosis fungoides
31	Male	66	90	90	90	20	CD7	Mutated	WT	Mutated	4	1	1	1	1	Mycosis fungoides
32	Male	20	95	95	95	30	None	WT	WT	WT	4	2	2	0	0	Mycosis fungoides
33	Female	50	95	95	95	20	None	Mutated	WT	WT	4	2	1	0	1	Mycosis fungoides
34	Female	46	80	90	90	10	None	Mutated	WT	WT	4	1	1	1	1	Mycosis fungoides
35	Female	23	80	90	80	40	None	Mutated	WT	WT	4	2	1	0	1	Mycosis fungoides

36	Female	38	95	95	95	5	None	Mutated	WT	WT	4	2	0	1	1	Mycosis fungoides
37	Male	42	95	95	95	20	CD7	Mutated	WT	Mutated	5	2	1	1	1	Mycosis fungoides
38	Female	29	90	95	90	40	None	Mutated	WT	Mutated	5	2	2	0	1	Mycosis fungoides
39	Male	12	60	95	40	3	None	Mutated	WT	Mutated	5	2	1	1	1	Mycosis fungoides
40	Female	68	95	95	95	30	None	WT	WT	Mutated	5	2	2	0	1	Mycosis fungoides
41	Male	68	95	95	80	5	CD7	Mutated	WT	Mutated	5	1	2	1	1	Mycosis fungoides
42	Female	34	95	95	80	20	None	Mutated	Mutated	WT	5	2	2	0	1	Mycosis fungoides
43	Female	46	90	95	80	60	None	Mutated	WT	Mutated	5	2	2	0	1	Mycosis fungoides
44	Male	36	90	90	90	50	None	Mutated	WT	WT	5	2	2	0	1	Mycosis fungoides
45	Male	37	80	95	95	5	None	Mutated	WT	WT	5	2	1	1	1	Mycosis fungoides
46	Male	11	95	95	70	3	CD7	Mutated	Mutated	WT	5	2	1	1	1	Mycosis fungoides
47	Female	35	90	95	90	3	CD7	WT	WT	Mutated	5	2	1	1	1	Mycosis fungoides
48	Female	61	20	30	20	3	None	Mutated	WT	WT	5	2	1	1	1	Mycosis fungoides
49	Male	26	95	95	95	15	None	Mutated	WT	WT	5	2	2	0	1	Mycosis fungoides
50	Female	29	90	90	60	20	CD5	Mutated	WT	WT	5	2	1	1	1	Mycosis fungoides
51	Female	54	85	85	55	20	CD7	Mutated	WT	WT	5	1	2	1	1	Mycosis fungoides
52	Male	17	70	95	90	5	CD7	WT	Mutated	Mutated	6	2	2	1	1	Mycosis fungoides
53	Male	39	80	95	70	5	None	Mutated	Mutated	Mutated	6	2	2	1	1	Mycosis fungoides
54	Male	24	95	95	60	5	None	Mutated	WT	Mutated	6	2	2	1	1	Mycosis fungoides
55	Male	51	5	95	5	5	None	Mutated	Mutated	Mutated	6	2	2	1	1	Mycosis fungoides
56	Male	56	90	95	90	5	CD5 & CD7	Mutated	WT	WT	6	2	2	1	1	Mycosis fungoides
57	Male	55	60	90	90	3	None	Mutated	WT	WT	6	2	2	1	1	Mycosis fungoides
58	Female	55	90	95	95	5	CD7	Mutated	WT	Mutated	6	2	2	1	1	Mycosis fungoides
59	Male	70	95	95	95	20	CD7	Mutated	WT	WT	6	2	2	1	1	Mycosis fungoides
60	Female	20	95	90	90	10	CD7	Mutated	WT	WT	6	2	2	1	1	Mycosis fungoides

Note: WT, wild-type.

Supplementary Table S2. Curvilinear coordinates of ROC, sensitivity, specificity, and Youden's index for CD2, CD3, CD5, and CD7 expression

Variable	Criterion (%)	Sensitivity	1 - Specificity	Specificity	Youden's index
CD2	4	0	0	1	0
	12.5	0.026	0	1	0.026
	40	0.053	0	1	0.053
	65	0.105	0	1	0.105
	75	0.132	0	1	0.132
	82.5	0.289	0.136	0.864	0.153
	87.5	0.316	0.136	0.864	0.18
	92.5	0.605	0.318	0.682	0.287
	97.5	1	0.955	0.045	0.045
	101	1	1	0	0
CD3	29	0	0	1	0
	55	0.026	0	1	0.026
	82.5	0.026	0.045	0.955	-0.019
	87.5	0.053	0.045	0.955	0.008
	92.5	0.289	0.227	0.773	0.062
	96	1	1	0	0
CD5	4	0	0	1	0
	12.5	0.026	0	1	0.026
	30	0.053	0	1	0.053
	47.5	0.079	0	1	0.079
	57.5	0.105	0	1	0.105
	65	0.158	0	1	0.158
	75	0.237	0	1	0.237
	85	0.342	0.136	0.864	0.206
	92.5	0.658	0.273	0.727	0.385
	97.5	1	0.955	0.045	0.045
	101	1	1	0	0
CD7	2	0	0	1	0
	4	0.158	0	1	0.158
	7.5	0.474	0	1	0.474
	12.5	0.526	0	1	0.526
	17.5	0.553	0	1	0.553
	22.5	0.737	0.227	0.773	0.51
	27.5	0.737	0.318	0.682	0.419
	32.5	0.816	0.409	0.591	0.407
	37.5	0.842	0.5	0.5	0.342

	45	0.921	0.591	0.409	0.33
	52.5	0.947	0.727	0.273	0.22
	57.5	0.974	0.773	0.227	0.201
	70	1	0.864	0.136	0.136
	82.5	1	0.909	0.091	0.091
	90	1	0.955	0.045	0.045
	96	1	1	0	0
